# How experience shapes individuality

**DOI:** 10.7554/eLife.112851

**Published:** 2026-09-11

**Authors:** Bassem A Hassan

**Affiliations:** 1 Paris Brain Institute Paris France; 2 Yale School of Medicine New Haven United States

**Keywords:** behaviour, genes, learning, individuality, environment, *D. melanogaster*

## Abstract

Behavioural diversity across fruit flies changes with individual learning, even when genetic, past and momentary environmental factors are held constant.

**Related research article** Manna R, Brea J, Braga GV, Modirshanechi A, Tomić I, Jakšić AM. 2026. Learning is a fundamental source of individuality. *eLife* **15**:RP111235. doi: 10.7554/eLife.111235.

No two individuals are absolutely identical. This is as true of behaviour as of morphology, and it is as true of fruit flies as of humans. Behavioural individuality is defined as a unique and persistent pattern of behaviour over time in one individual that differs from that of other individuals in the same population. Individual variation in behaviour has many origins, with genetic variation thought to be a major one (Plomin, 2023; Dudai et al., 1976).

However, decades of research across animal species have demonstrated that even in populations and species with almost no genetic variation, behavioural individuality persists and is no less variable than in populations with genetic diversity. Another obvious source of behavioural individuality is environmental variation. Yet under experimental conditions where both life history and the experienced environment are tightly controlled, genetically identical populations continue to show behavioural individuality, suggesting an important contribution from intrinsic differences between individuals (Linneweber et al., 2020).

Another key contributor to individuality is, of course, environmental experience. The environment varies unpredictably over time, and animals learn from experience, generalise what they learn, and adjust their behaviour accordingly (Kandel and Hawkins, 1992). Two models are plausible for how the environment might contribute to behavioural variation. A changing environment may uniformly affect behaviour across a population. If so, the effect would be expected to be the same for individuals that learn and for those that do not. In this case, the distribution of variation in the population would not be expected to change as the environment changes. Conversely, and perhaps more interestingly, learning from a changing environment may affect individuals differently, thereby contributing to behavioural individuality within a population over time. Whether and how this learning “on the fly” (pun intended) actually contributes to shaping behavioural individuality was unknown.

Now, in eLife, Ana Marija Jakšić and colleagues at the École Polytechnique Fédérale de Lausanne, Lausanne– including Riddha Manna as first author– report that individual learning experience furthers behavioural variability beyond the effects of past events (Manna et al., 2026). To overcome the challenge of controlling for genetics, life history, and learned experience across a large number of individuals and learning episodes, Manna et al. turned to the tiny but mighty fruit fly as a model system.

In a tour de force study, they designed a visual learning test in which flies were punished with mild electric shocks for initially randomly choosing to enter a blue tunnel versus a green tunnel, or vice versa (Figure 1). Exploiting known natural variation in intrinsic colour preference in flies (Anderson et al., 2017), Manna et al. designed the experiment so that the fly would receive a shock before or after making its initial decision, depending on its initial colour bias in the non-learning choice experiment. This clever design was used to examine thousands of individual flies from 90 different isogenic populations, performing nearly half a million behavioural episodes under conditions where flies either learn or do not learn rules about a changing environment. Next, the researchers turned to computational modelling to confirm that individual learning specifically contributed to the variance in behavioural individuality – regardless of genetics, the environment itself and life history.

**Figure 1.**
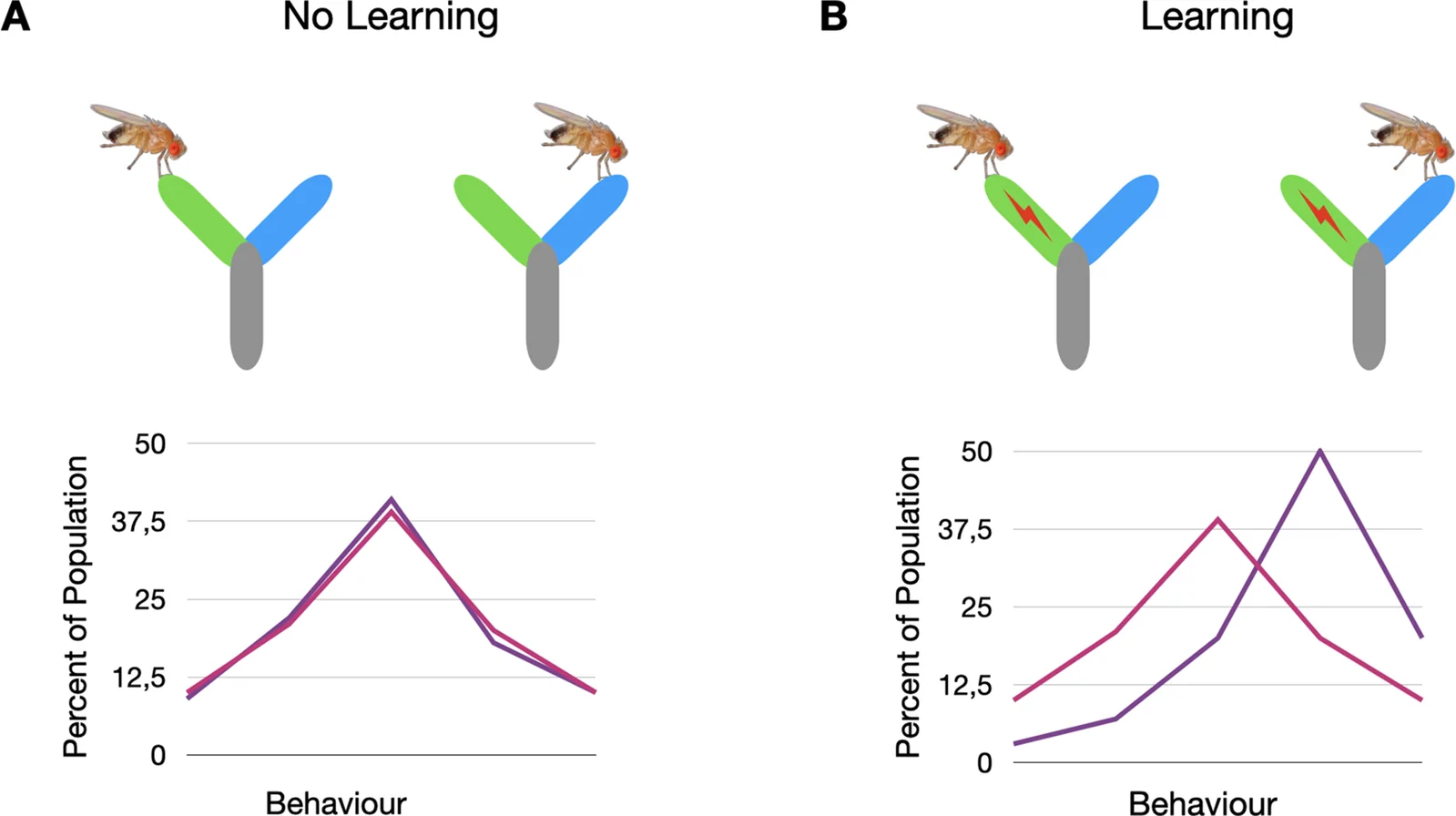
Learning shapes behavioural individuality. Flies are allowed to choose between entering a blue tunnel or a green tunnel in a Y-maze. (**A**) Under control conditions, the initial random choice has no consequences for the fly and therefore no specific association between the behaviour. Here, the behavioural distribution of the population (pink graph) does not change after exposure to the Y-maze (violet graph). (**B**) In contrast, when one of the two colours is punished by a mild electric shock, flies learn to associate their initial choice with a negative consequence. Manna et al. discovered that this not only alters the future behaviour of individuals, but also the distribution of this individuality across the population.

The main conclusions from this study are as exciting as they are unexpected. First, Manna et al. provide quite compelling evidence for the role of moment-by-moment learning episodes in diversifying behavioural individuality: the distribution of variation changes as individuals learn. Second, bias in the first decision an animal makes during a learning episode changes the trajectory of its future learning curve and individualised behaviour. This suggests that stochastic intrinsic variation interacts with stochastic learning variation to continually diversify individuality: the biases the flies have before learning affect how they learn and how they apply this knowledge, providing a rigorous scientific demonstration of the famous “butterfly effect” (Lorenz, 1963). Finally, they show that computational modelling of these complex interactions at the individual animal level is possible.

This work underscores the critical importance of understanding behaviour as an emergent property of interactions among underlying stochastic elements, and thus of analysing and comparing it within and across populations, rather than treating it merely as an average property of a population. This concept harkens back to Ernst Mayr’s “population thinking” (Mayr, 1959), which rejects population averages and emphasises the critical importance of population distributions and individual variance as key to evolutionary processes, and thus to life itself.

Like any good scientific study, this work raises as many questions as it answers. How persistent over time, and how general across sensory modalities, is the effect of momentary learning on individual behaviour? How predictable are individuals’ initial random choices, given the combination of their genetic variation, developmental trajectories and experience? Finally, what neural processes underlie behavioural diversification arising from momentary learning? Model organisms such as fruit flies, where all of these features are accessible to carefully controlled experimental manipulation, will no doubt continue to play a key role in answering those fundamental questions. This may provide inroads to a better understanding of complex human decision-making. A need that appears urgent at a time when humanity is facing multiple crises of its own making, not least the existential threat of global warming.
